# Seeing the unseen? Illusory causal filling in FIFA referees, players, and novices

**DOI:** 10.1186/s41235-016-0008-5

**Published:** 2016-09-22

**Authors:** Alisa Brockhoff, Markus Huff, Annika Maurer, Frank Papenmeier

**Affiliations:** Department of Psychology, Eberhard Karls Universität Tübingen, Schleichstr. 4, Tübingen, 72076 Germany

**Keywords:** Expertise influences, Event perception, Dynamic scenes, Causal fillings

## Abstract

Humans often falsely report having seen a causal link between two dynamic scenes if the second scene depicts a valid logical consequence of the initial scene. As an example, a video clip shows someone kicking a ball including the ball flying. Even if the video clip omitted the moment of contact (i.e., the causal link), participants falsely report having seen this moment. In the current study, we explored the interplay of cognitive-perceptual expertise and event perception by measuring the false-alarm rates of three groups with differing interests in football (soccer in North America) (novices, players, and FIFA referees). We used the event-completion paradigm with video footage of a real football match, presenting either complete clips or incomplete clips (i.e., with the contact moment omitted). Either a causally linked scene or an incoherent scene followed a cut in the incomplete videos. Causally linked scenes induced false recognitions in all three groups: although the ball contact moment was not presented, participants indicated that they had seen the contact as frequently when it was absent as in the complete condition. In a second experiment, we asked the novices to detect the ball contact moment when it was either visible or not and when it was either followed by a causally or non-causally linked scene. Here, instead of presenting pictures of the clip, the participants were give a two-alternative forced-choice task: “Yes, contact was visible”, or “No, contact was not visible”. The results of Experiment 1 indicate that conceptual interpretations of simple events are independent of expertise: there were no top-down effects on perception. Participants in Experiment 2 detected the ball contact moment significantly more often correctly in the non-causal than in the causal conditions, indicating that the effect observed in Experiment 1 was not due to a possibly influential design (e.g., inducing a false memory for the presented pictures). The theoretical as well as the practical implications are discussed.

**Electronic supplementary material:**

The online version of this article (doi:10.1186/s41235-016-0008-5) contains supplementary material, which is available to authorized users.

## Significance

The current work is, to our knowledge, the first to combine a study of perceptual-cognitive skills with event perception and it is, therefore, mainly of an explorative nature. We took theoretical research out into the real world and investigated the role of top-down factors on event completion by testing three groups with a differing level of interest and experience (novices, players, and FIFA referees) on a simple event-completion task (Strickland &amp; Keil, 2011). Although there is considerable evidence that expertise in sports domains is connected to superior perceptual-cognitive skills, our results indicate no influence of these skills on event perception. They rather support a recent publication by Firestone and Scholl (2015b), who concluded that perception may be largely independent of top-down influences. Such a proposition not only challenges our theoretical understanding of event perception, but also has substantive practical implications for fairness in sports by strongly advocating the increased use of technology instead of perceptual training programs for match officials.

## Background

During the FIFA World Cup tournament in 2010, the referees made many controversial calls that influenced the outcomes of matches so tremendously that the then-FIFA president apologized for the referees’ mistakes. In response, the use of goal-line technologies was officially allowed in 2012, which since have become more and more common at the very top levels of the game. The current study was inspired by a controversial goal that happened in a Bundesliga match in 2013, a match in which no goal-line technology was used. The ball went through a hole in the side netting and everyone, including the referees, mistook it for an actual goal. This rare phantom goal demonstrated the limits and biases of human perception. Such a phantom goal is even more surprising in the light of numerous studies that reported experts to have superior domain-specific perceptual-cognitive skills (e.g., Williams, 2000), an expertise that even leads to an advantage in motion outside the expert’s area (e.g., Romeas & Faubert, 2015). Vision and perception are shaped by one’s individual experiences and knowledge: the mental representations of events. Such representations are reconstructed and updated through experience and knowledge and provide the basis for understanding the world around us (Zacks & Tversky, 2001). However, constant reconstruction and updating of mental representations make event perception effortful and, thus, fragile. Strickland and Keil (2011) reported a (possibly consequential) bias in event perception: the event-completion effect. Video clips that indicated a causal implication (example sequence: an athlete running towards a ball – cut – a flying ball) produced higher false-alarm rates for pictures displaying the athlete kicking the ball than video clips that did not imply any causation. The authors suggested that observers either confused online predictions (the ball will be kicked and will bounce down the field) with actually seen elements of the scene, or relied on schema- or principle-based post hoc inferences (a ball bouncing down a field must have been kicked).

***Perceptual-cognitive expertise*** A number of studies have reported that expert athletes show superior perceptual-cognitive skills compared to novices in sport-specific tasks, including visual cue usage (Abernethy, Gill, Parks, & Packer, 2001; Ward, Williams, & Bennett, 2002; Williams, 2000), visual search strategies (Vaeyens, Lenoir, Williams, & Philippaerts, 2007; Williams, 2000), and recall and recognition of meaningful patterns (Bell, Boshuizen, Scherpbier, & Dornan, 2009; Lesgold et al., 1988; Reingold & Sheridan, 2011; Smeeton, Ward, & Williams, 2004). In general, experts’ demonstration of perceptual-cognitive expertise can go beyond the specific sports domain (Romeas & Faubert, 2015; Romeas, Guldner, & Faubert, 2016) and can help, for example, in learning complex neutral dynamic scenes (Faubert, 2013) or to outperform novices in everyday tasks (e.g., crossing a street as a pedestrian in a crowded inner city: Chaddock, Neider, Voss, Gaspar, & Kramer, 2011). While the majority of the reported studies intended to identify the exceptional perceptual-cognitive skills of experts by focusing on pattern recognition, decision-making, or biological motion perception, mainly aiming to create training programs or prevent incidents that result in injuries, the current paper is interested in a fundamental understanding of experts’ perception, or memory, of events.

***Hypotheses*** In the current study, we conceptually replicated the design by Strickland and Keil (2011) and tested two expert groups (football players and FIFA referees) and a control group (students with no interest in football). We wondered whether the perceptual-cognitive skills of experts would prevent the event-completion effect when observing familiar motion. Based on the currently most prominent model of event perception, the event segmentation theory (EST; Zacks, Speer, Swallow, Braver, & Reynolds, 2007), prediction errors occur and an event boundary is perceived when certain event features change (e.g., situational features such as spatial location and characters: Zacks, Speer, & Reynolds, 2009). If online predictions of experts are more detailed, it may be more likely that the missing ball contact is actually reported to be perceived as a missing situational feature in the schema, and, thus, not perceptually filled in. More specifically, a more detailed representation would result in a lower false-alarm rate in referees and players.

We do have reason to hypothesize that the superior perceptual-cognitive skills of experts could prevent the event-completion effect since they may process visual information not only qualitatively but also quantitatively differently, but the opposite could be the case as well. Mann, Williams, Ward, and Janelle (2007) analyzed eye movements of experts and novices and revealed that the skilled performers required fewer fixations of longer duration to gather relevant information, compared to novices, who made many short fixations. Thus, novices consider the potential influence of all available visual information while experts concentrate on the relevant information by perceiving the multidimensional complexity of the situation (further examples are in Haider & Frensch, 1996; Hattie, 2003; North & Williams, 2008). Expertise was also shown to allow for a more efficient switch of attentional foci. Underwood, Chapman, Brocklehurst, Underwood, and Crundall (2003) observed that the scan paths during driving differ depending on the expertise of the driver. Novices were not able to switch their focus of attention as a response to potential hazards, while experts constantly monitored other road users. In the current study, the hardwired event schemata of experts could actually lead to a stronger bias if the ball contact is considered irrelevant information in the representation of the event. Or stated differently, novices may have a more detailed schema of the event (e.g., a ball kick) because, in their lives, there is no need for them to condense the schema for more efficient processing. Referees, however, have to make 3 or 4 decisions in each minute of the actual play time (Williams, 2013) and, thus, they benefit significantly from filtering visual information rigorously. On the other hand, experts may have a more detailed schema than a novice due to frequent exposure and the ability to switch their focus of attention if needed. However, based on the EST, this again would result in a stronger event-completion effect. If experts have a rather global observational approach to familiar scenes, they may even have event models that account for missing information and changes in visual information. The missing ball contact may then not be surprising; therefore, it may not be detected as an error, and will, thus, not result in the perception of an event boundary but in an event-completion effect. Finally, it is also possible that there are simply no top-down effects of cognition on perception as recently claimed by Firestone and Scholl (2015b). The two authors carefully reviewed hundreds of studies and extracted general (design) pitfalls of each approach to study the effect of cognition on attention. We will discuss our results with regard to the two disparate but interrelated systems of perception and memory.

***Experimental overview***

To ensure that we really tested perceptual-cognitive differences in event perception – and not declarative knowledge and analysis skills – we intentionally used video clips of dynamic events that did not require knowledge of the game, depicting actions that definitely have been observed by each participant before, independent of their level of interest in football. We cut out scenes from a real match, including corner kicks, kick-offs, free kicks, and throw-ins. In Experiment 1, we conceptually replicated the design of Strickland and Keil (2011) and presented the participants with (1) the complete sequences (i.e., including the contact moment), (2) an incomplete causal sequence (i.e., excluding the ball contact), or (3) an incomplete non-causal sequence (i.e., excluding the ball contact with a non-logical follow-up; example sequence: player about to throw the ball in – cut – a different player being fouled). However, note that our restricted sample of experts did not allow us to run a between-subject design as was done in the original study. To ensure that our design would not alert the participants to the purpose of the event, we left out one condition: a visible ball contact that was followed by a non-causal scene. In Experiment 2, we further controlled the design by showing video clips that either included or excluded a ball contact. Participants were fully informed about the probabilities of each clip type occurring (50 %) and were given a forced choice of the two alternatives (ball contact seen: yes or no). The latter inevitably brought in the aspect of attentional control by “knowing what to look for”; however, it helped us to understand further at which point of information processing the bias has its origin. We are aware, however, that our (or any) design may not be able to grasp the fine line between perception, memory, and post-perceptual judgment. Our results will be discussed with a focus on the event-completion effect and its occurrence in different groups. Any interpretation concerning perception or memory has to be regarded with caution.

## Methods

**Fig. 1:**
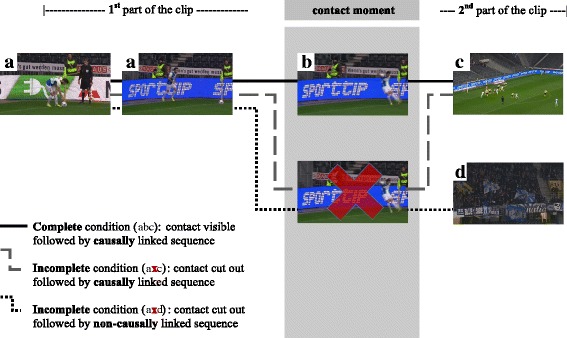
Example sequences (pictures). **a** First part of the clip. **b** Ball release/contact moment. **c** Second part of the clip: causally linked scenes. **d** Second part of the clip: not causally linked scenes

Stimuli were presented on 15.4-inch notebooks using PsychPy (Peirce, 2008). The participants were seated at a distance of 60 cm from the screen. Footage of a soccer match of the Young Boys Bern against the Grasshoppers Zürich that took place on 23 March 2014 was used as stimulus material. The footage was compiled out of three camera perspectives. Clips of about 20 seconds each were created. Each clip consisted of two parts shot from different camera angles. The assignment of clips to conditions was balanced across participants in each experiment. In general, the two parts of each clip were causally linked or not (Fig. 1 c or d), and the ball release or contact (kick) moment^1^ (Fig. 1 b) was visible or not. Figure 1 depicts example sequences.

**Fig. 2:**
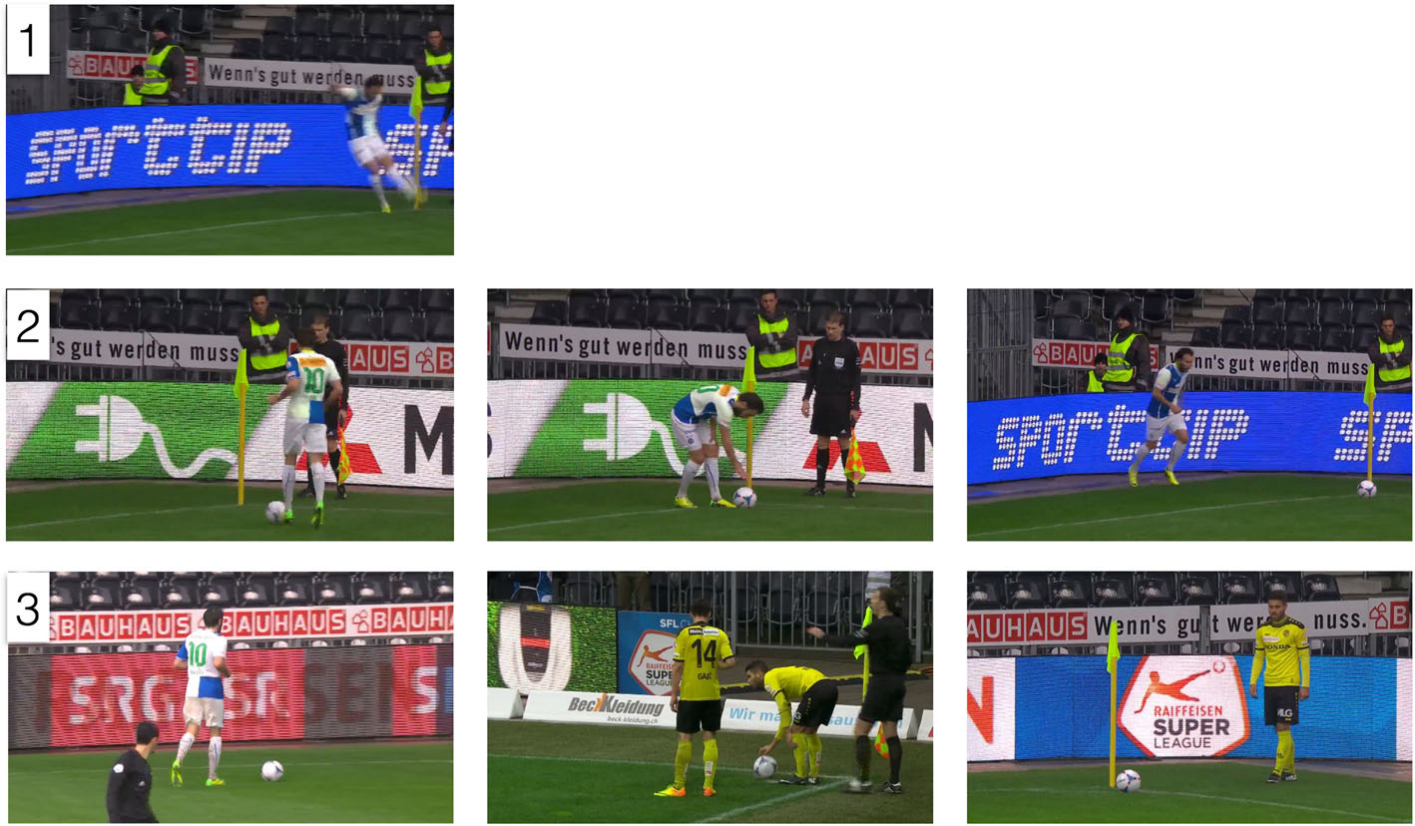
Example response pictures. 1: Contact, 2: yes filler (selected from the first part of the clip), 3: no filler (not in the clip)

In Experiment 1, we conceptually replicated the design by Strickland and Keil (2011) and used the following combinations of video clips (see Fig. 1): complete (A–B–C) vs incomplete causal (A–C) vs incomplete non-causal (A–D).^2^ In Experiment 2, the basic idea of the design was similar; however, we measured only the detection rate of the contact moment and further added a condition in which the ball contact (B) was visible in non-causal sequences as well (A–B–D). In Experiment 1, each participant saw seven response pictures (see Strickland & Keil, 2011) after each clip. Three pictures were selected from the first part of each clip (a yes filler), three pictures were related to the yes-filler items but came from other parts of the game, such as other players preparing for a corner kick (a no filler), and the critical picture depicted the moment of ball contact or ball release (contact). The participants were asked whether they had seen the picture in the clip: Yes (“press 1”) or No (“press 9”). See Fig. 2 for the response pictures for the example sequences (Fig. 1). Further, they were asked to rate how certain they were about their answer (on a scale from 1, not at all, to 5, extremely).

In Experiment 2, we showed the participants 40 clips and asked whether they had seen the ball contact moment (B in Fig. 1). Instead of response pictures (Fig. 2), we gave the participants forced-choice alternatives: “Yes, I have seen the ball contact” and “No, I have not seen the ball contact”. The experiment was conducted as a mixed 2 (ball contact visible, within) × 2 (second part of the clip: causal or non-causal, between) subject design. We measured the sensitivity to the contact moments as *d* ^′^ and response criterion *c* (see Experiment 2 for further details).^3^

An expertise questionnaire tested basic declarative football knowledge using 11 questions, for example, “In which country did the last FIFA World Cup take place?” (see Additional file 1: Appendix for a complete list of questions).

***Statistical analysis***

In Experiment 1, we report expertise knowledge, proportion correct, proportion of yes answers, and confidence in the recognition test as separate dependent variables. Because of the binary response variable (yes or no), we analyzed effects on *proportion correct* and *proportion of yes answers* with a generalized mixed effect model (with a logit link), using the lme4 package (Bates, Sarkar, Bates, & Matrix, 2007; Pinheiro, Bates, DebRoy, & Sarkar, 2006) in the R environment (R Development Core Team, 2016). Participants were specified as the random factor to control for their associated intraclass correlation. We present the type II Wald *χ* ^2^ test results from GLMER. Further, we provide the results of planned contrasts (based on our hypotheses and the original study’s results). Additionally, the credibility of the found null effect and the likelihood of the occurrence of the null and the alternative hypotheses are presented with Bayesian statistics and JASP (JASP Team, 2016). In Experiment 2, we report the sensitivity measure *d* ^′^.

### Experiment 1: Conceptual replication of the original study with groups with different expertise levels

#### Method

**Participants** Three groups of participants were tested on three different occasions. There were 42 novices (14 male and 28 female students, age *M* = 25.76, SD = 6.81 years), 16 football players of a seventh German football league (all male, age *M* = 24.81, SD = 3.64 years), and 18 referees from Switzerland appointed as officials for matches in competitions organized by the Fédération Internationale de Football Association (FIFA) (all male, age *M* = 32.2, SD = 4.93 years). Two referees were excluded because they retired from their active positions as official FIFA referees. The students tested participated in return for monetary compensation or course credits. The football players were students of the University of Tuebingen’s department of sports science and their participation was a course requirement. The referees participated during one of their regular advanced training courses and were not compensated monetarily.

**Design and procedure** The first part of each clip was between 11.6 and 15.1 seconds long. A keeper during a kick-off was depicted in three clips, a throw-in in one clip, a corner kick in three clips, and a free kick in two clips. A clip was either shown completely or shortened by the removal of the moment of ball contact (kick) or ball release (throw-in). We deleted 1–4 frames; however, the deletion for causal and non-causal clips was always exactly the same. The second part of the clip lasted between 5.7 and 8.4 seconds. Each participant saw nine clips spread equally across three conditions: complete first part with causally linked second part (complete), shortened first part with causally linked second part (incomplete with causally linked sequence), or shortened first part with second part that was not causally linked (incomplete with non-causally linked sequence). See Fig. 1, combinations A–B–C, A–C, and A–D. The experiment reported here took 15 minutes. The participants received instructions and immediately started with the event-completion task. After each clip, seven response pictures (Strickland & Keil, 2011) were shown (see Fig. 2).

#### Results

**Expertise knowledge** We calculated the proportion of correctly answered questions. The football players’ declarative football knowledge was significantly higher compared to the novices’ (*M* =.86, SD =.34 and *M* =.51, SD =.50, respectively): *t*(50.83)=10.70, *p* &lt;.001. We regarded the referees’ football knowledge as a precondition for their FIFA employment and did not test them on the questionnaire.

**Fig. 3:**
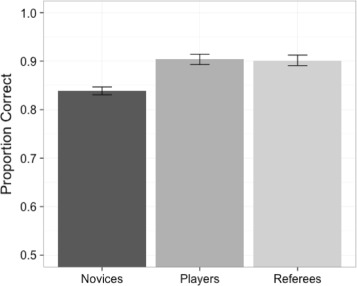
Performance in the recognition test (excluding the critical contact item) as a function of expertise. Error bars represent the standard error of the mean

**Proportion correct** We analyzed participants’ performance in the recognition test. Because the critical contact item was a target item in the complete condition and a distractor item in the remaining two conditions, we excluded this item from this analysis. We calculated the proportion of correctly answered questions and fitted a generalized mixed effect model with the binary dependent variable *yes/no answers*. Expertise was inserted as the fixed effect, and participants were specified as the random factor. The factor expertise was significant [ *χ* ^2^(2) = 17.621 and *p* &lt;.01]. Post-hoc Tukey comparisons helped to specify the difference between the three groups of expertise. As can be seen in Fig. 3, the two expert groups outperformed the novices: for players vs novices *z* = 3.33 and *p* &lt;.01, and for referees vs novices *z* = 3.254 and *p* &lt;.01. We observed no differences between the players’ and the referees’ performance (*z* = 0.06, *p* =.99).

**Proportion of yes answers.** We analyzed the effects on the binary dependent variable (yes/no answers) with a generalized linear mixed model (with a logit link), using the lme4 package in the R environment. Participants were specified as a random factor to control for their associated intraclass correlation. We used the raw data and fitted a model including all main effects and interactions of expertise, item type, and condition as fixed effects. We analyzed the resulting model using type II Wald *χ* ^2^ tests.

**Fig. 4:**
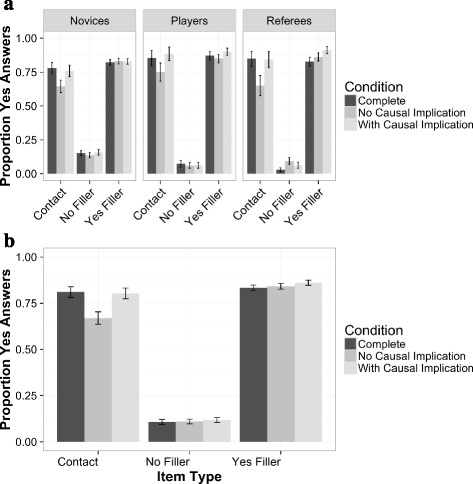
Proportion of yes answers. **a** Proportion of yes answers for each expertise level as a function of condition (complete, no causal implication, with causal implication). **b** Aggregated proportions of yes answers as used in the contrast analysis. Error bars represent the standard error of the mean

Our main finding is a significant two-way interaction of condition and item type [ *χ* ^2^(4)=11.52 and *p* =.021]. The three-way interaction of expertise, condition, and item type was not significant [ *χ* ^2^(8)=6.91 and *p* =.546]. Further, there was a significant main effect of item type [ *χ* ^2^(2)=1262.00 and *p* &lt;.001], and a significant interaction of expertise and item type [ *χ* ^2^(4)=41.05 and *p* &lt;.001]. None of the other main effects and interactions reached significance,*p*&gt;.17. While the proportion of yes answers in the non-causal condition was significantly lower (as expected), it should be noted that the false-alarm rate was still over chance level. However, our findings are in line with the results found in the original study by Strickland and Keil (Strickland and Keil 2011). See Fig. 4 a for the analyzed proportions in each expertise group.

To investigate the interactive relationship of the two categorical variables *condition* and *item type*, we calculated contrasts. The underlying *glmer* model was now reduced (see Fig. 4 b for the aggregated data used) and did not include expertise anymore, since the given expertise level (novice, player, or referee) did not interact with condition*item type (non-significant three-way interaction reported above). To prevent *α* inflation at this level of the analysis, a Bonferroni correction (0.05/3=0.016) for multiple comparisons was applied. Further insights into the variability of the (log) mean difference between the observed answers are given with 95 % confidence intervals (CI).

As expected, two of the three contrasts produced significant results. The number of yes answers (i.e., the number of reports indicating that the contact moment had been seen) in the condition with implied causation (causal) differed significantly (*z* = 22.21 and *p* &lt;.001) from the number of yes answers in the condition *without* implied condition (non-causal), with an estimated (log) mean difference of 4.03, CI [3.60, 4.46]. The non-causal incomplete condition also differed significantly from the condition in which the ball contact was included (complete condition), *z* = 16.51 and *p* &lt;.001 (estimated difference = 3.95, CI [3.52, 4.38]). The contrast of the causal vs the complete condition was not significant, *z* = 0.73 and *p* = 0.75 (estimated difference = 0.15, CI [ −0.36, 0.68]).

**Bayesian statistics** We calculated a Bayes factor analysis for the proportion of yes answers to the contact items in the no causal implication and the conditions with causal implication. The Bayes factor evidence for the null hypothesis in a Bayesian repeated measures ANOVA comparing a model that included the main effects of condition (with causal implication or no causal implication) and expertise (novices, players, and referees) with a model including additionally the interaction of these factors amounted to 4.99, which is conventionally classified as substantial (Rouder, Morey, Speckman, & Province, 2012; Wetzels & Wagenmakers, 2012).

**Fig. 5:**
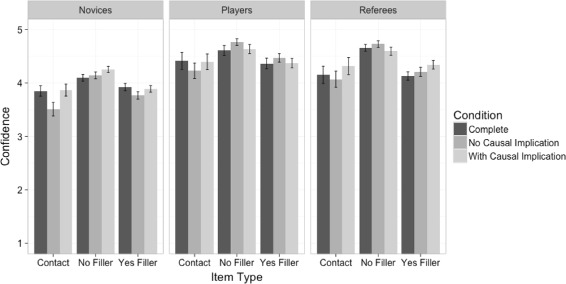
Confidence rating as a function of condition and expertise. Error bars represent the standard error of the mean

**Confidence** A repeated measures ANOVA was performed with confidence as the dependent variable (see Fig. 5). We observed a significant main effect of expertise [ *F*(2,71)=10.27 and *p* &lt;.001]. Players’ and referees’ confidence was significantly higher than novices’ confidence,*p*&lt;.004. Again, there was no difference between players and referees (*p* =.501). Further, we observed a significant main effect of item type [*F*(2,142) = 27.20 and *p* &lt;.001], indicating that confidence was higher for the no-filler items compared to the contact items and the yes-filler items,*p*&lt;.003. The interaction of item type and condition approached significance [*F*(2,142) = 2.42 and *p* =.049]. In this context, however, we observed no significant differences between the different conditions with regard to the contact item responses, *p*&gt;=.247.

#### Discussion

To capture online perceptual performance errors, we presented video clips that implied causation (or not) and asked the participants afterwards whether they had seen certain pictures (or not). While overall performance (proportion correct) was higher for experts than for novices, all participants were prone to the event-completion effect (analyzed with the proportion of yes answers). Furthermore, we measured confidence rating to examine whether experts show illusionary superiority biases (observed as a coping mechanism for stress and self-esteem protection in referees; e.g., Wolfson & Neave, 2007). We observed higher confidence ratings in the referee and the player groups compared to the novices – however, they actually performed better, thus, showing an actual superiority instead of an illusionary superiority bias. This was expected based on the experts’ superior recall and recognition of meaningful patterns and details (Bell et al., 2009; Lesgold et al., 1988; Reingold & Sheridan, 2011; Smeeton et al., 2004). The results of the present study replicate the event-completion effect measured in the original study by Strickland and Keil (2011). The results exemplify how the human information processing system struggles with perceiving and recalling details of an everyday life event. We found these difficulties to be independent of task-specific expertise, suggesting that on a certain basic perceptual level, if presented with a simple action event, humans equally chunk or segment continuous activity, resulting in the representation of a series of discrete events (Newtson, 1973) – a process that allows for online and post-hoc inferences, and illusory causal fillings. However, before we interpret these results further, we need to ensure that the effect found is not due to the study instructions, which may have biased the participants to assume ball contact. Participants may have *assumed* they had seen contact because they did not know that omitted contact moments were an option.

The question remains whether the observed event-completion effect is a phenomenon based on online predictions or rather the result of backwards mapping, an effect known from text comprehension research (e.g., Potts, Keenan, & Golding, 1988). Although, backwards mapping was originally used to explain anticipation processes during text comprehension, its adaption to causal fillings in event perception is straightforward: participants base their decisions of a recognition item at the very moment of presentation and check if the picture is a plausible cause of what they have already watched. In other words, a contact picture would be a plausible, and natural, cause of a video clip that showed a football player approaching a ball.

### Experiment 2: contact – yes or no?

In a detection experiment, we presented participants with complete and incomplete stimuli with causal and non-causal continuation and asked them to indicate whether they had seen the contact moment or not. This may prevent backwards mapping because participants “know what to look for” before the presentation of the video clip. Further, without recognition items (pictures), the participants are less prone to picture-based biases, which allows us to measure participants’ discrimination performance in the non-causal and causal conditions. If the event-completion effect is primarily a phenomenon based on online predictions, participants’ discrimination performance should be lower in the causal compared to the non-causal condition.

#### Method

**Participants** Altogether, 32 students of the University of Tuebingen (7 male and 25 female students, age *M* = 23.16 years, SD = 4.61) participated in the experiment in return for course credits or monetary compensation. Of these, 17 participants were assigned to the causal and 15 to the non-causal condition. We excluded from the analysis one participant who did not understand the task. Thus, 17 in the causal and 14 participants in the non-causal condition entered the final analysis.

**Design and procedure** Then 40 video clips were shown either as complete clips or with the ball contact excluded. The first part of each clip was between 1.4 and 15 seconds long. The clips were either causally connected or not (see “General method”). We always deleted four frames before the ball contact frame, resulting in a deletion of 160 ms in both incomplete conditions (the presentation rate of each clip was 25 frames per second). The second part of the clip was between 1.2 and 6.3 seconds long.^4^ Clips consisted of 14 kick-offs, 5 corners, 13 throw-ins, and 8 free kicks. Participants received specific information on the probability that the ball contact was visible (50 %). Further, they saw a process graphic of a matchstick man approaching a ball and kicking it so that they knew what “ball contact moment” or “moment of ball release” meant. The suggested experiment has been conducted as a mixed 2 (ball contact visible, within-subject manipulation) × 2 (second part of the clip: causal or non-causal, order was balanced between groups) design.

#### Results

We report sensitivity (*d* ^′^) and response criterion (*c*) from signal detection theory as dependent variables (Green & Swets, 1966). Yes answers to clips depicting the release moment (complete conditions) were counted as hits and yes answers to clips not depicting the release moment (incomplete condition) were counted as false alarms. Finally, we aggregated the data on the participant level and calculated separate independent sample *t*-tests for *d* ^′^ and *c*. Because *d* ^′^ and *c* are not defined for hit rates and false-alarm rates of 1.0 and 0.0, we adjusted such values to half a trial incorrect or half a trial correct, respectively.

**Sensitivity** Sensitivity (*d* ^′^) was well above chance (*d* ^′^=0) and was significantly higher in the non-causal (*M*=2.75, SD = 0.59) compared to the causal condition (*M* = 1.44, SD = 1.15), *t*(29) = 4.08, *p* &lt;.001. Thus, this supports the hypothesis that participants’ online perception was distorted by the causal continuation of the scene.

**Response bias** We did not observe a statistically significant difference between the non-causal (*M*=−0.09, SD = 0.32) and the causal condition (*M*=−0.20, SD = 0.38) with regard to the response criterion (*c*), *t*(29) = 0.88 and *p* =.388.

#### Discussion

We observed lower discrimination performance in the group of participants who saw the causal sequel compared to the group of participants who were presented with non-causal sequences. Thus, these findings support the hypothesis that the causal continuation actually changed participants’ perceptions. A (cautious) explanation of this finding refers to EST (Zacks et al., 2007). According to EST, participants’ perceptions are based on predictions. For a non-causal continuation, these predictions fail and participants perceive an event boundary. As a consequence, participants’ representations of this moment are more precise compared to the condition with non-causal continuation in which predictions were not violated and participants did not perceive an event boundary.

### General discussion

The present study was interested in the interplay of cognitive and perceptual processes in experts compared to novices. The main objective was to study the appearance of the event-completion effect in groups with different cognitive-perceptual training. However, our results also give us an idea of how internal schema-based systems and external sensory input processing may result in an automatic completion of events. The results reported here allow a number of interesting implications.

#### Theoretical implications for event perception

The current most prominent model of event perception is EST (Zacks et al., 2007). EST is based on various theories of perception, neurophysiology, and language processing (Carpenter, Grossberg, & Arbib, 2003; Fuster, 1990; Van Dijk, Kintsch, & Van Dijk, 1983). A fundamental principle of EST states that the processing of events forms sensory representations that are influenced by experience and knowledge. Event schemata affect the current content of the event models with top-down processes, expanding their effective capacity by assembling predictive information about the future relevance of certain features of events. When certain event features change (e.g., situational features such as spatial location and characters; Zacks et al., 2009), prediction errors occur and an event boundary is perceived. Regarding EST, our results could be explained with an error-detection mechanism that operates on a temporal buffer holding a given number of causal snapshots (Wood, 2011). The error-detection mechanism constantly checks whether online predictions based on working memory representations of the ongoing event are fulfilled. Transient increases in the violation of predictions (Zacks et al., 2009) make the current event model useless and in need of an update. As our results suggest, one missing snapshot of an event (implied causation condition) does not automatically trigger an event update because enough predictions of the event are fulfilled. Clip sequences that did not imply causality may have activated an error-detection mechanism and triggered event boundary perception processes. The original EST model describes event models as a stable representation that can only be reset or updated based on the current perceptual information available when the error-detection mechanism opens the gate. Error detection may also play an important part in the actual perception of events: the comparable number of yes answers for contact items and causal yes-filler items in our data implies that the event-completion effect is nurtured by the sensitivity of the error-detection mechanism. In other words, the more prediction errors the error monitoring allows, the more illusory causal fillings will happen. Importantly, our data suggest that expertise does not influence event perception. That indicates that top-down processes do not influence the simple mechanisms of online prediction and error detection as much as is assumed in the EST (Zacks et al., 2007). This top-down component, however, is largely underspecified in EST. Zacks and colleagues write: “This claim is based largely on parsimony and may need to be revised in the future” (Zacks et al., 2007, p. 275). At least for our stimulus material with simple structured events, the idea of an unaffected gating mechanism is in line with Firestone and Scholl (2015b): there are no top-down effects of cognition on perception.

#### Top-down effects and the locus of contextual biases

Did the participants in our studies actually see (falsely perceive) or did they simply report to have seen (falsely remember) the ball contact? The presented studies applied a recognition and detection test to explore the event-completion effect. However, as recently suggested by Firestone and Scholl (in press), there is a great difference between seeing and recognizing. Any top-down effect measured can be due to an influence on front-end visual processing but equally likely be due to back-end memory. In the current paper, we communicated a tendency to define the event-completion effect as due to an error that occurs in perception rather than in memory. Although we do not have clear evidence for either involvement, the results of Experiment 2 (in which we decreased the possible memory biases due to backwards mapping) do indicate that the effect is partly due to online perceptional processes. We were further biased by the majority of results found in the literature that connect memory to experience. As memory fades due to brain damage or aging, representations become increasingly changed by preexisting knowledge. Especially popular is that patients with Alzheimer’s tend to falsely remember details, words, or events that they actually did not experience (confabulation: e.g., Tallberg & Almkvist, 2001). However, experience and expertise did not influence the appearance of the event-completion effect. Thus, reversing the argument, our results could show that the event-completion effect cannot be an error in memory, because then we would have found differences between the expertise groups.

In a recent paper (Firestone & Scholl, 2015a), the authors discuss semantic (language) priming, universally understood as an effect on memory (Collins & Loftus, 1975) that may have been mistaken for top-down effects on visual processing in various studies. In semantic priming, reading a word such as “peach” lowers the threshold for related fruits in memory and they will be processed faster than an unrelated word. Language and event perception are closely related: much of what we know about our understanding of events comes from studies that asked participants to describe an event in their own words. For example, with such a linguistic account, Talmy (1975) was able to define the building blocks of motion events. However, it may be possible that the observed language structure does not only reflect how we perceive event units, but could be a general reflection of the preferred global-over-local approach of the human brain (e.g., Fink et al., 1996). If we assume that the activation of related words is comparable to the activation of related event models in memory (allowing for faster access to different scenarios and faster processing of related visual details), our null findings would again point towards a bias on the perceptual level. The wealth of experienced scenarios of the event should have activated a broader spectrum of experienced content in the experts, which should have resulted in differences between the three groups due to differences in memory activations. Firestone and Scholl (2015a) further proposed that it is possible to distinguish memory and perception clearly in practice. This seems to be a bold proposal since false memories (here, an error of commission) can be elicited within 1/20th of a second (Intraub & Dickinson, 2008). Intraub and Dickinson (2008) report a constructive error in scene representation, the boundary extension, in which observers falsely remember an image that is shown beyond the edges of the previously encountered view. When the first item is presented without a scenic structure, boundary extension does not occur (Intraub, Gottesman, & Bills, 1998). They propose that boundary extension is the result of a source-monitoring error (Johnson, Hashtroudi, & Lindsay, 1993) with a strong influence of a reality-monitoring error (Johnson & Raye, 1981). The error happens when the human brain has to distinguish between internally generated information (experience with certain structures) and externally generated information (sensory input). The authors suggest that the rapidity of such a boundary extension error is advantageous rather than harmful; it shows how the visual system incorporates fleeting views of images with spurious boundaries into a coherent representation of the world around us. The rapidity of the error may further imply that perception and memory are two processing systems of the same underlying cognitive mechanism.

Our data could be explained as the result of a distinction error between internally and externally generated information. Disregarding the traditional distinction of false memories and visual illusions and assuming an extraordinary fast engagement of both during the processing of visual input, the observed null effect of expertise in our experiment may be the result of an imbalance of weighted sources. The externally generated information processing of experts may be more efficient and more detailed; however, the internally generated information outperforms sensory input due to the system’s need to embed the event into known reality. Experiment 2 further reflects the weight of the reality source. Here, participants knew precisely what would be tested in each trial and were prepared to answer a specific question. Conscious awareness is needed to be able to report whether the stimulus was visible or not (Lamme, 2003), but even in such an enhanced state of target processing, the internally generated source overruled the external sensory input, resulting in decreased sensitivity for the detection of the ball contact moment in causally linked scenes. For the current design, the ideas mentioned above are pure speculations and may be regarded as such. Future research could be concerned with whether expertise influences the level (global or local) of event processing. For example, Beaucousin et al. (2011) recorded event-related potentials and reported that the meaningfulness of an object influences global and local information. They assumed that knowledge about the world influences the global and local levels of processing. Comparing the performance of experts and novices on meaningful and non-meaningful patterns would help us to understand better the early stages of processing.

In addition, it would be interesting to see whether experts compared to novices structure events differently, measured as event segments indicated with a button press by participants. As memory distortions can happen within 50 ms (Intraub & Dickinson, 2008), behavioral measurements may not be able to grasp the difference between memory and perception (if there is any). To really answer such a question, functional neuroimaging procedures are advisable.

#### Practical implications

The present findings have a serious impact on the fairness of the game. A red card may be based simply on two single observations that perceptual processes have falsely interconnected in a causal manner: player A approached player B and player B got hurt. The match official may be absolutely certain that they had seen a contact, but it may have been an event-completion effect. The top Dutch football league (*Eredivisie*), therefore, employs a video referee who observes video replays of the game to help the referees on the field with tricky decisions. However, since many believe that the human element of sports is lost when technologies are used, eliminating, for example, the “enjoyment of debating mistakes” (Kelso, 2010), chances are rather low that other European football leagues will follow the example of the Dutch. Even in the presence of technology, the importance of the perceptual and cognitive skills of match officials is, thus, not reduced.

#### Limitations

It needs to be taken into account, however, that we aimed to test basic perceptual processes and can, thus, speak only about the organization of the mind when it is faced with simple events. The perception of complex events may nonetheless be influenced by domain-specific expertise. For example, when presented with a deliberate dive, novices may not be able to differentiate between whether it was a real foul or a fake fall by the player. The cognitive-perceptual excellence and the so-called intuitive skills of an expert to analyze such an incident may be based on a highly sensitive error-detection mechanism. Such ideas, however, will require theoretical and empirical development beyond the scope of this article. Left unknown is still whether memory or perception is responsible for the effect.

## Conclusions

In conclusion, the results of the present study demonstrated a short-cut of the human information-processing system to deal with missing information when faced with causally linked video sequences: the event-completion effect. We explored basic processes that may be biased by an imbalance in external and internal source weighing, based on the similarity found for three groups of expertise. This indicates that the influence of higher cognitive processes in the observation of simple action events may be overruled by the human need to make sense of the world and the need to embed an event into a known structure.

Bearing in mind that we tested referees who had achieved the highest qualification level and who officiated at international FIFA matches, it is fair to surmise that the event-completion effect for simple events is hardwired. Perceptual training programs that focus on *external sensory input* to prevent causal fillings of events will be difficult to design. Finally, the observed effect illustrates impressively that, without further game technology in the future, football players, fans, coaches, and journalists do not have to worry about losing the drama and the thrill of being defrauded by the human brain’s biases.

## Endnotes

^1^ Note that we use the term contact moment throughout the rest of the paper to refer to both the kicking and releasing of the ball.

^2^ Note that we present only one experiment event even though there were two. However, the hypotheses and the design were completely unrelated to the goal of this work and will be analyzed and published independently.

^3^ Note that a between-subject design was reported in the original study but was not feasible in Experiment 1 due to the small sample of referees and time constraints during testing. In Experiment 2, we asked students to participate in the laboratory. Thus, using a between-design was possible and, additionally, allowed us to ensure that participants could not guess the purpose of the experiment when seeing both critical conditions.

^4^ Note that the length of the second part of the clip was a natural consequence of the events happening in the footage of the match and not an intentional manipulation.

## Additional file

Additional file 1 Appendix. (DOCX 85.6 kb)

## Abbreviations

CI: confidence intervals
EST: Event segmentation theory
