# Supplementary material for: Seeing the unseen? Illusory causal filling in FIFA referees, players, and novices
Source: Cogn Res Princ Implic. 2016 Sep 22;1:7. doi: 10.1186/s41235-016-0008-5 (PMC5256435; doi:10.1186/s41235-016-0008-5)
Supplement: Supplementary file 1 — Appendix. (DOCX 85.6 kb) [file 41235_2016_8_MOESM1_ESM.docx]

**Appendix**

No. of participant. _______

**We want to know how well-informed you are about football and how much you are interested in this game!**

**Therefore, we would like you to answer a couple questions about football.**

**Have fun!**

**Please mark the right answers with a cross or write your answer in the space below.**

1. What is a cross?
   1. A high pass into the opposing penalty area/ in front of the opposing goal
   2. A pass from the left sports field to the right
   3. A long pass from behind to the front
2. How many teams are in the German premier league?
3. In which case is a player in an offside position?
   1. When the player at the moment the ball is played by a teammate is nearer to the opponent's goal than at least two opponents.
   2. When the player at the moment the ball is played by a teammate is nearer to the opponent's goal than at least one opponent.
   3. When the player at the moment the ball is played by a teammate is nearer to the opponent's goal than the goalkeeper.
   4. When the player leaves the sports field.
4. In which country did the last FIFA World Cup take place?
5. What is the meaning of a ''dive'' ?
   1. A player falls over to get a free kick
   2. A player jumps high over an opponent
   3. A player complains vehemently
6. Where is the goalkeeper allowed to touch the ball with his hands?
   1. In the five-meter area
   2. In the sixteen-meter area
   3. In the seven-meter area
7. What is a „Sechser“?
   1. A central midfielder
   2. A central defender
   3. A central striker
8. When does an indirect kick occur?
   1. On a handball
   2. On an offside
   3. On a goal-kick
   4. On a foul
9. Where was the first FIFA World Cup?
   1. Spain
   2. Germany
   3. Uruguay
   4. Russia
10. Which country is record-breaking world champion?
11. What is a “back-four formation”?
    1. A forward-line about four players
    2. A midfield-line about four players
    3. A defensive-line about four players

Do you have any comments regarding this experiment?

**The experiment is over. Please inform the examiner that you are done!**
